# Bat pollinators: a decade of monitoring reveals declining visitation rates for some species in Thailand

**DOI:** 10.1186/s40851-024-00228-x

**Published:** 2024-03-02

**Authors:** Alyssa B. Stewart, Supawan Srilopan, Kanuengnit Wayo, Piriya Hassa, Michele R. Dudash, Sara Bumrungsri

**Affiliations:** 1 Department of Plant Science, Faculty of Science, Mahidol University, Bangkok, Thailand; 2 Department of Biology, Prince of Songkla University, Hat Yai, Songkhla, Thailand; 3 Department of Natural Resource Management, South Dakota State University, Brookings, SD USA; 4 Department of Biology, University of Maryland, College Park, MD USA

**Keywords:** Biodiversity, Chiropterophily, Conservation, *Eonycteris*, *Macroglossus*, Pollination, Pteropodidae

## Abstract

Bats are important pollinators, but they are difficult to study since they are volant and nocturnal. Thus, long-term studies of nectarivorous bats are scarce, despite their potential to help assess trends in bat populations and their pollination services. We used capture rates of nectarivorous bats at chiropterophilous flowers in order to examine temporal trends in bat visitation in an area that is undergoing extensive land use change. We mist-netted at five bat-pollinated plant taxa (*Durio zibethinus*, *Musa acuminata*, *Oroxylum indicum*, *Parkia speciosa*, and *Sonneratia* spp.) in southern Thailand over six years between 2011 and 2021. We found that the most common bat species, *Eonycteris spelaea*, was the main visitor at all five plant taxa and had consistent visitation rates across all study years. In contrast, two other important pollinators, *Macroglossus minimus* and *M. sobrinus*, showed 80% declines in the number of individuals netted at mangrove apple (*Sonneratia* spp.) and banana (*Musa acuminata*) flowers, respectively. These findings suggest that *E. spelaea* (a large, cave-roosting species with a broad diet) is more tolerant of anthropogenic change than are *Macroglossus* bats (small, foliage-roosting species with specialized diets), which may in turn affect the reproductive success of plants pollinated by these species. Our study demonstrates how decade-long monitoring can reveal species-specific temporal patterns in pollinator visitation, emphasizing the need for tailored conservation plans. While the conservation status of most nectarivorous bats in the area is Least Concern, our results indicate that population studies in Southeast Asia are urgently needed for updated bat species conservation assessments.

**Supplementary Information:**

The online version contains supplementary material available at 10.1186/s40851-024-00228-x.

## Background

While most pollination research has focused on bees and other insects [53, 72], bats are highly important pollinators for many night-blooming plant species [19, 35]. Bat-pollinated plants are often highly specialized [59] and typically share little overlap with other pollinator groups [67]. Previous research has shown that vertebrate-pollinated plants are more dependent on their pollinators than are insect-pollinated plants, especially in the tropics [50]. Furthermore, bat-pollinated plants are more dependent on their pollinators than plants that are pollinated by other vertebrate groups, such as birds or rodents [50]. Many of these bat-pollinated plant species are ecologically and economically important [35, 60].

Despite their importance, bat pollinators are understudied, in part, because of the difficulty of studying these elusive, nocturnal, and volant animals [32]. Pollinating bats in the paleotropics present an even greater challenge, given that they do not echolocate, which precludes the use of acoustic monitoring and requires capturing individuals in order to identify them to species [23]. Thus, because they require large investments in time, energy, and funding, long-term ecological studies of bats (i.e., > 10 years sensu [32]) are scarce (but see [25, 28, 33, 47, 65]). However, multi-year studies can be informative for assessing trends in bat populations and their pollination services [23, 28, 32], especially given how quickly landscapes are changing in the Anthropocene [58, 74].

Most research examining the effects of anthropogenic stressors on pollinators has focused on insects, but bats are affected by many of the same challenges. For example, large-scale studies of insect pollinators have found that land-use type and intensity affect pollinator diversity, especially in tropical regions [42], and especially for pollinators with narrow diets [72]. Moreover, fragmentation [4, 26] and climate change [20, 49] can also negatively affect insect pollinators. Research examining how bat pollinators respond to anthropogenic change is less comprehensive, but studies published to date have highlighted similar overall patterns. Habitat loss and degradation are some of the biggest threats to bats [23, 31, 41, 46]. Similarly, Regan et al. [52] found that habitat loss due to agriculture is one of the main drivers of extinction among mammalian pollinators, most of which are bats, and that pollinating bat species tend to be more threatened than non-pollinating bat species. Moreover, flower-visiting bats exhibit dietary shifts in response to changes in landscape structure [56] and climate change can affect the distributions of nectarivorous bats and the plant species they pollinate, resulting in spatial mismatch between plants and pollinators [75]. One of the key findings among many of these studies is that the effect of anthropogenic disturbance varies by region and pollinator species [27, 41, 42, 53, 72]. Thus, research is still needed for many pollinator taxa, such as understudied bat pollinators, and multi-year studies can provide valuable information about how pollinators are responding to anthropogenic changes.

In particular, previous research has stressed the need for more bat research in understudied tropical areas such as Southeast Asia [23, 34, 41, 48]. We conducted a decade-long study on the pollinating bats of southern Thailand, collecting data in 2011–2014 and 2019–2021. The flora of this region includes many species that are pollinated by bats, including the economically important durian (*Durio zibethinus* L. [13]) and a critically endangered mangrove species (*Sonneratia griffithii* Kurz [45]). Much is known about the ecology of the flower-visiting bat species in the area [1–3, 10–13, 54, 56, 60–63], but we lack reliable information about their population sizes and trends. For example, the IUCN Red List [30] classifies many of the local flower-visiting bat species as Least Concern, but the lack of concrete information makes such assessments untenable. Inconsistent with these IUCN assessments, a study in Singapore estimated that 33–72% of the country’s bat species are now locally extinct, and projected that at least 23% of the bat species in Southeast Asia will be extinct by 2100 [36]. Moreover, the study area has changed substantially over the past few decades. A recent study demonstrated that, between 1995 and 2015, southern Thailand lost 21% of terrestrial forests, 26.2% of mangrove forests, and 55% of peat swamp forests, with up to 33% of remaining forests classified as highly vulnerable to future land conversion [64]. Given such extensive land-use changes, we examined bat capture rates at key chiropterophilous plant species in southern Thailand between 2011–2021 in order to assess trends in bat populations and the pollination services they provide.

## Methods

### Study area

This work was conducted in southern Thailand (Phatthalung, Satun, Songkhla, and Trang provinces; 6°32'–7°36' N, 99°21'–100°37' E) where nectarivorous bats and bat-pollinated plant species are common [63]. The area is dominated by rubber and oil palm plantations intermixed with other agricultural landscapes (e.g., rice paddies and fruit orchards), patches of natural habitat (e.g., lowland tropical forests and mangrove forests), and human settlements [60, 64, 68]. The climate is tropical. The average minimum temperature in our study area is 24^o^C, the average maximum temperature is 34°C, and the average yearly precipitation is 1,800 mm (years 1991–2020) (Climatological Center, Thai Meteorological Department,www.climate.tmd.go.th).

### Study species

The most common flower-visiting bat species in our study area include three nectar-specialist bat species (*Eonycteris spelaea* (Dobson, 1871); *Macroglossus minimus* (E. Geoffroy, 1810); and *M. sobrinus* K. Andersen, 1911) and four primarily frugivorous bat species (*Cynopterus brachyotis* (Müller, 1838); *C. horsfieldii* Gray, 1843; *C. sphinx* (Vahl, 1797), and *Rousettus leschenaultii* (Desmarest, 1820)) [63]. The nectar-specialist species have long muzzles and tongues characteristic of nectarivores [22], and forage almost exclusively on floral resources [11, 40, 62, 63]. In contrast, the primarily frugivorous species have powerful jaws and well-developed molars [21], and while they mainly forage on fruits, they have also been observed foraging at flowers [12, 40, 63].

**Fig. 1:**
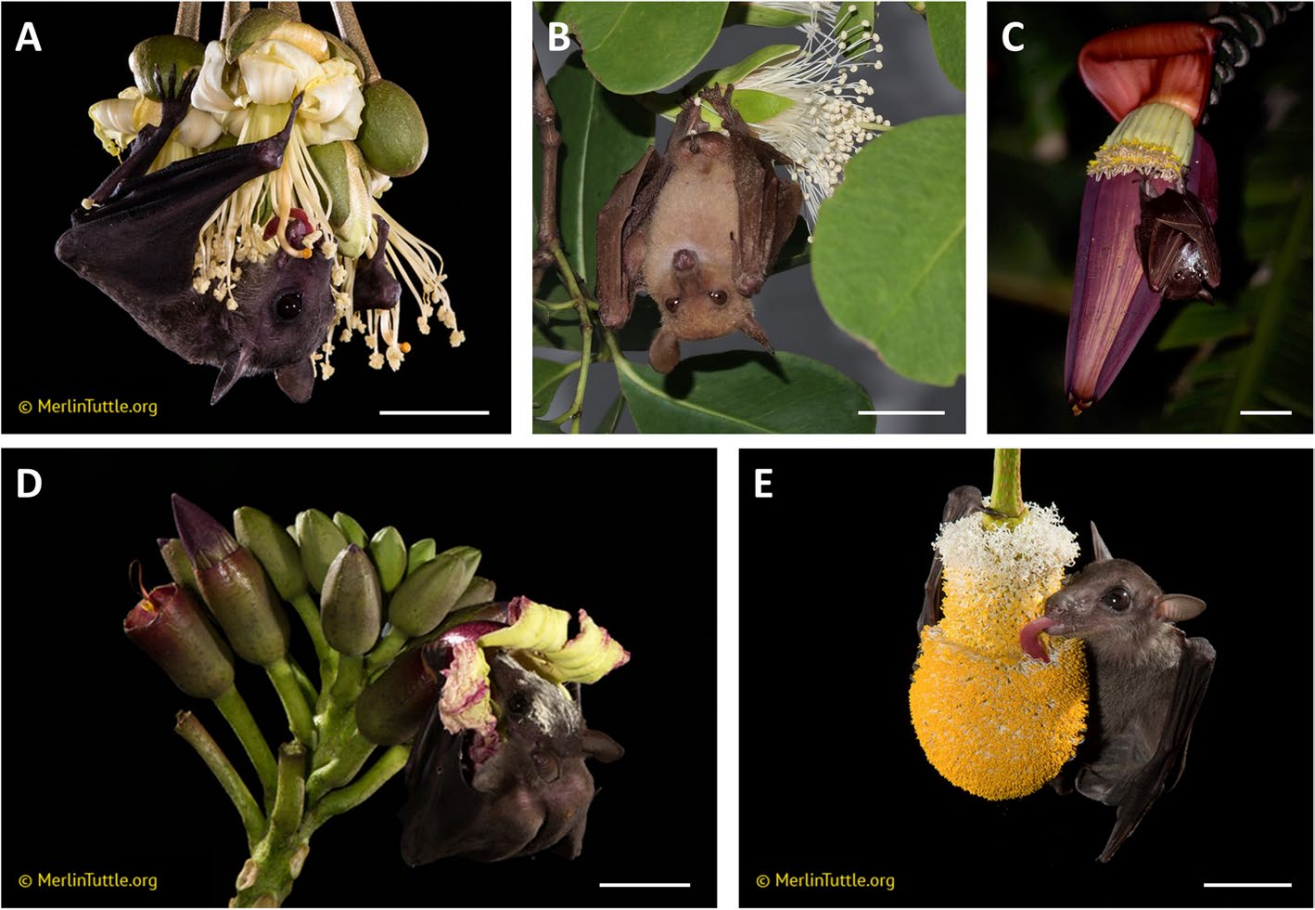
Plant study species with their main pollinators. **A** *Durio zibethinus* inflorescence with *Eonycteris spelaea*, (**B**) *Sonneratia alba* flower with *Macroglossus minimus*, (**C**) *Musa acuminata* inflorescence with *Macroglossus sobrinus*, (**D**) *Oroxylum indicum* inflorescence with *Eonycteris spelaea*, and (**E**) *Parkia speciosa* inflorescence with *Eonycteris spelaea*. White scale bars represent 3 cm. Photos A, D, and E were taken by Merlin Tuttle, photos B and C were taken by Alyssa Stewart

We focused on five bat-pollinated plant taxa for this study: *Durio zibethinus* L., *Musa acuminata* Colla, *Oroxylum indicum* (L.) Benth. ex Kurz, *Parkia speciosa* Hassk., and *Sonneratia* L.f. species (Fig. 1). These species are some of the major food resources for nectar-feeding bats [11, 60, 62]. *Durio zibethinus* (Malvaceae, durian) is an economically-important fruit crop in the region that exhibits mass flowering, producing several thousand flowers in the span of about 10 days [13]. Flowers are hermaphroditic and, depending on the cultivar, can be either self-incompatible or self-compatible [13, 69]. *Musa acuminata* (Musaceae, banana) is a temporally dioecious, herbaceous plant species ubiquitous throughout southeast Asia [6, 29]. Each shoot produces a single inflorescence that displays 15–40 flowers per night for multiple weeks [29], and flowering individuals can be found year-round [24]. While cultivated bananas are parthenocarpic, wild plants require pollination to set fruit [5, 6]. *Oroxylum indicum* (Bignoniaceae, Indian trumpet flower) is a self-incompatible tree species found throughout much of Asia [54]. Flowers are hermaphroditic and only a few open per night, but flowering trees can be found year-round [55]. *Parkia speciosa* (Fabaceae, sator or petai) is a self-incompatible tree species that can have up to 70 capitula open per night [10]. Capitula contain 2,500–4,000 flowers, and inflorescences are either hermaphroditic or functionally staminate [10]. *Sonneratia* (Lythraceae, mangrove apple) is a paleotropical mangrove genus with hermaphroditic flowers and flowering tends to occur in flushes [57, 66]. Four species of *Sonneratia* are found in our study area (*S. alba* Sm., *S. caseolaris* (L.) Engl., *S. griffithii* Kurz, and *S. ovata* Backer, [60].

### Data collection

In order to assess the frequency of each bat species at each of our plant study species, we used mist-nets (Avinet Research Supplies, Maine, USA) to catch foraging bats. We mist-netted at our plant study species for a total of 23 nights in 2011, 34 nights in 2013, 34 nights in 2014, 23 nights in 2019, 26 nights in 2020, and 20 nights in 2021 (4–10 nights per plant species per year; Supplementary Table 1). We changed mist-netting locations each night to minimize avoidance learning. Mist-netting sites differed across years but tended to be in the same general areas (e.g., netting at different houses or farms in the same village). For each plant species, we mist-netted when flowers were highly abundant (between March and May for *D. zibethinus*, between May and October for all other study species). For two plant species we were only able to collect data in four years of the study period (*D. zibethinus*: 2013, 2014, 2020, 2021; *Sonneratia*: 2013, 2014, 2019, 2020). Mist-nets were placed as close as possible to open flowers (from 18.30 – 24.00 h) and netting heights were similar across years (Supplementary Table 1). Nets were checked for bats every 15–30 minutes. Bat captures were used to determine the overall number of bats netted per hour (total number of bats netted divided by the total number of mist-net hours; net size: 6 x 2.6 m) for each bat species at each focal plant species. Netted bats were identified to species following Francis [21], held in breathable cloth bags to prevent repeat captures, provided with sugar water, and released after mist-nets were taken down.

### Statistical analysis

All analyses were performed in R 4.2.2 [51]. To examine trends in bat visitation throughout our study period, we examined bat capture rates at each plant species across years using linear mixed modeling (LMM, function *lmer*, package “lme4”; [8]). Separate analyses were conducted for each focal plant species. We included capture rate (bats per hour) as the response variable; bat species, year, and their interaction as fixed factors; and site as a random factor. Factor significance was assessed using the *joint_test* function (package “emmeans”; [37]) and, when significant, factor levels were compared using Tukey’s post-hoc test (function *emmeans*, package “emmeans”; [37]) with a Holm-Bonferroni correction to control for multiple comparisons. We also compared bat capture rates across two time periods, pooling data collected in 2011–2014 and data collected in 2019–2021. The models were set up and analyzed in the same way as above, except that we used time period as a fixed factor instead of year. All graphs were created using the “ggplot2” package [71].

## Results

**Fig. 2:**
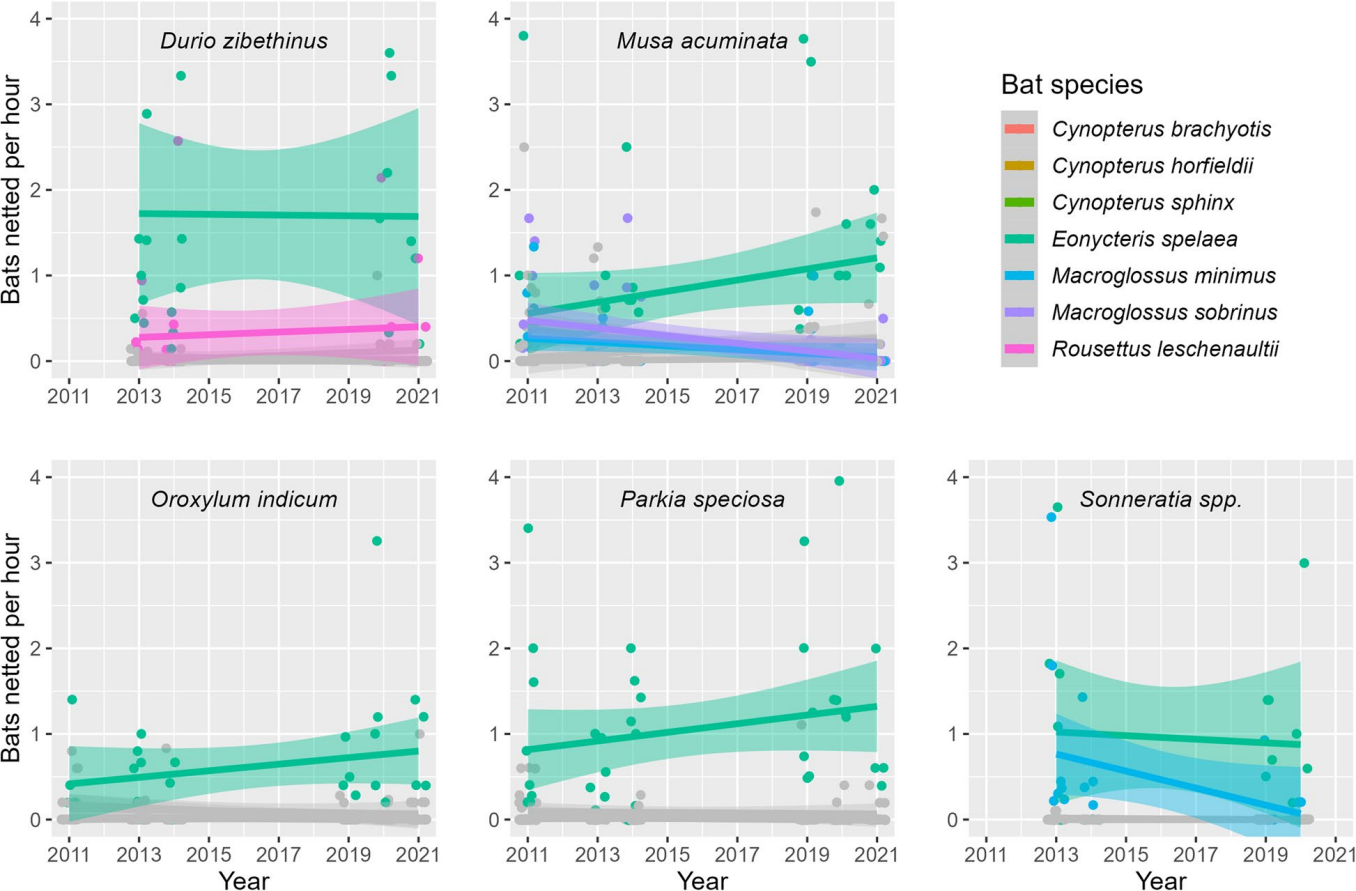
Results of LMM showing the number of bats netted per hour for seven flower-visiting bat species at five bat-pollinated plant taxa between 2011-–2021. Points show actual data; lines and shaded areas show linear regression lines and 95% confidence intervals, respectively. Note: Only data for the known pollinators of each plant taxa are shown in color, all other bat species are shown in grey; the full color figure is shown in Supplementary Figure 1.

LMM results revealed that capture rates at flowers differed among bat species at all five plant study species, but overall capture rates at each plant species did not change across years (Table 1). However, there was a significant interaction between bat species and year for banana (*M. acuminata*), Indian trumpet flower (*O. indicum*), and mangrove apple (*Sonneratia* spp.) (Table 1). At durian (*D. zibethinus*) flowers, *E. spelaea* was the most frequent visitor followed by *R. leschenaultii*, and both were netted significantly more often than the remaining bat species (Fig. 2; Supplementary Figure 1). At sator (*P. speciosa*) flowers, *E. spelaea* was the only regular visitor and was netted significantly more often than the remaining bat species (Fig. 2). For the other three plant taxa, post-hoc results were more complicated, given the significant interaction between bat species and year. At banana flowers, multiple bat species were common visitors in 2011-2014, including its known pollinators *E. spelaea* and *M. sobrinus* (Supplementary Figure 2). However, in 2019–2021, only *E. spelaea* was a regular visitor while *M. sobrinus* visits were rare (Supplementary Figure 2). At Indian trumpet flowers, *E. spelaea* was the dominant visitor across all years, while *C. horsfieldii* was a relatively common visitor in years 2011–2014 but was rarely netted in 2019–2021 (Supplementary Figure 2). At mangrove apple flowers, *E. spelaea* and *M. minimus* were equally dominant visitors in 2013–2014, while only *E. spelaea* was regularly netted in 2019–2020 (Supplementary Figure 2).

**Table 1:** Linear mixed model results (F-ratio, degrees of freedom (df), and *p*-values (P)) showing the effect of two main factors (bat species and year) and their interaction on the number of bats netted per hour at the flowers of five bat-pollinated plant taxa. Significant predictors are highlighted in bold

	Predictor	F-ratio	df	*P*
*Durio zibethinus*	Bat species × Year	0.775	6	0.591
	**Bat species**	41.62	6	<0.001
	Year	0.004	1	0.948
*Musa acuminata*	**Bat species × Year**	4.738	6	<0.001
	**Bat species**	22.34	6	<0.001
	Year	3.778	1	0.059
*Oroxylum indicum*	**Bat species × Year**	2.191	6	0.047
	**Bat species**	31.29	6	<0.001
	Year	0.358	1	0.555
*Parkia speciosa*	Bat species × Year	2.119	6	0.052
	**Bat species**	79.93	6	<0.001
	Year	1.153	1	0.290
*Sonneratia* spp.	**Bat species × Year**	4.867	6	<0.001
	**Bat species**	26.18	6	<0.001
	Year	19.59	1	0.382

**Fig. 3:**
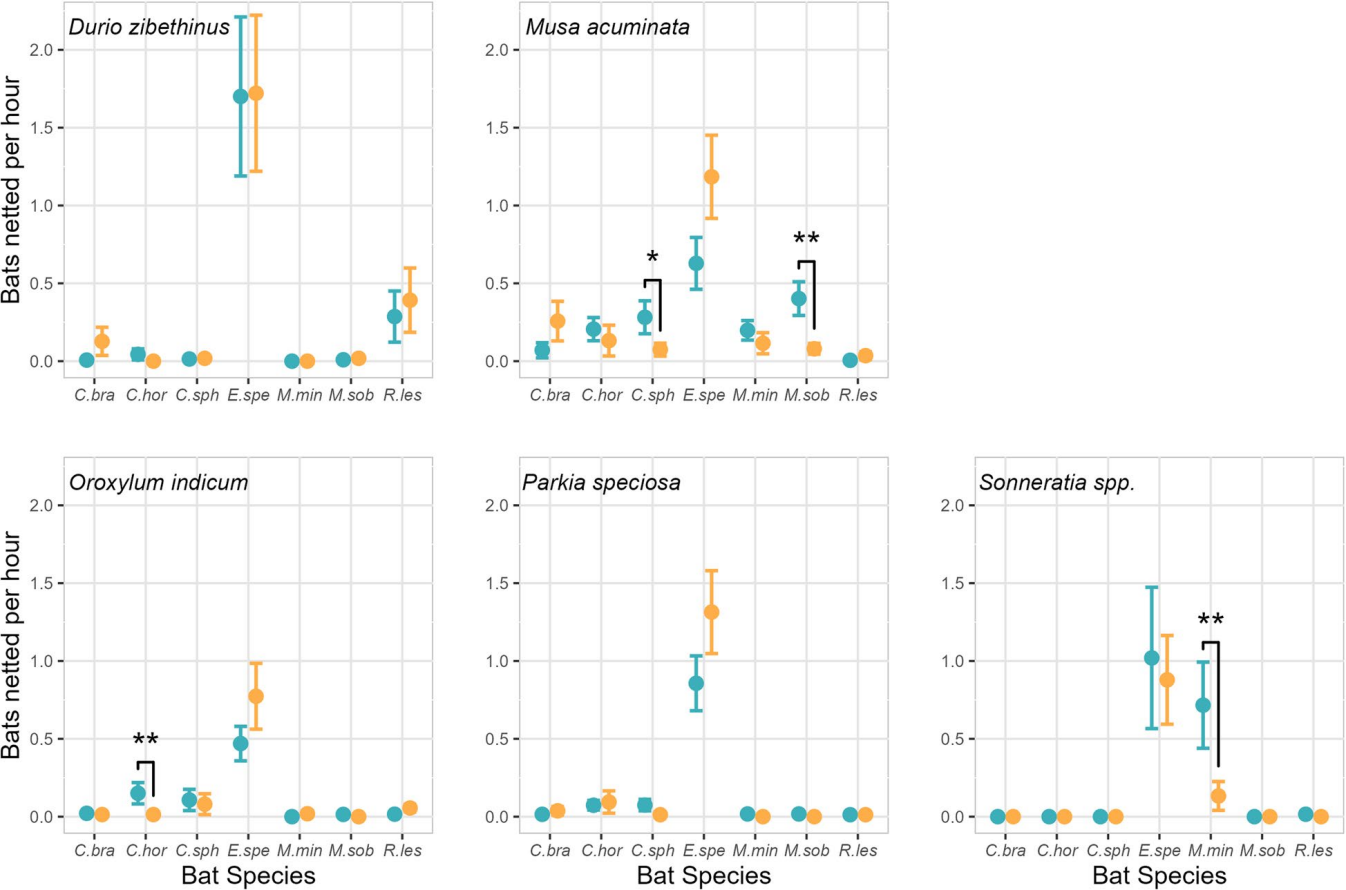
A comparison of the number of bats netted per hour (mean ± SE) for seven nectar-feeding bat species at five bat-pollinated plant taxa between 2011–2014 (teal) and 2019–2021 (orange). Significant differences between the two time periods are denoted with asterisks (one asterisk, *P* < 0.05; two asterisks, *P* < 0.01). Abbreviations: *C. bra*, *Cynopterus brachyotis*; *C. hor*, *Cynopterus horsfieldii*; *C. sph*, *Cynopterus sphinx*; *E. spe*, *Eonycteris spelaea*; *M. min*, *Macroglossus minimus*; *M. sob*, *Macroglossus sobrinus*; *R. les*, *Rousettus leschenaultii*

Comparing data pooled into two time periods (2011–2014 versus 2019–2021) via LMM revealed similar results: bat species was significantly different for all plant taxa, time period was not significant for any plant taxon, and the interaction between bat species and time period was significant for banana (*M. acuminata*), Indian trumpet flower (*O. indicum*), and mangrove apple (*Sonneratia* spp.) (Supplementary Table 2). For durian (*D. zibethinus*) and sator (*P. speciosa*) flowers, the number of bats netted for each bat species generally did not differ between the two periods (Fig. 3; Supplementary Figure 3). For banana flowers, the numbers of *M. sobrinus* and *C. sphinx* bats netted were significantly lower in 2019-2021 than in 2011-2014 (an 80.2% and a 73.8% decrease, respectively; Fig. 3; Supplementary Figure 3). For Indian trumpet flowers, the number of *C. horsfieldii* bats netted was significantly lower in 2019–2021 than in 2011–2014 (a 91.1% decrease; Fig. 3; Supplementary Figure 3). For mangrove apple flowers, the number of *M. minimus* bats netted was significantly lower in 2019–2021 than in 2011–2014 (an 81.4% decrease; Fig. 3; Supplementary Figure 3).

## Discussion

Between 2011 and 2021, we observed declines in the number of bats netted at floral resources for some bat species, with results varying by plant taxon. The strictly nectarivorous species, which are the main pollinators of chiropterophilous plants in southern Thailand [60], exhibit different patterns, with some species maintaining consistent visitation rates over the past decade and others exhibiting significant declines. The primarily frugivorous species had low visitation rates across all plant taxa and across all years, making it difficult to assess long-term patterns, and they contribute little towards the pollination of chiropterophilous plant species in southern Thailand [60]. Thus, we focus our discussion on the strictly nectarivorous species.

*Eonycteris spelaea* is an important pollinator of diverse plant species in southern Thailand [10, 11, 13, 54, 60], and the findings of this study demonstrate that the number of *E. spelaea* netted at five bat-pollinated plant taxa has remained consistent or even increased (though not significantly) over the past decade (Figs. 2, 3). These findings suggest that *E. spelaea* is relatively tolerant of the changes in land use that occurred in the study area (e.g., agricultural intensification and urbanization, [64]). Indeed, we commonly netted *E. spelaea* in banana and durian orchards, as well as at flowering plants next to houses and on university campuses. The ubiquity of *E. spelaea* across diverse anthropogenic habitats indicates that this species is relatively undisturbed by artificial lighting and human activity, which may be due to its relatively large size (45-75 g; [21], A. Stewart, pers. obs.) that potentially makes it less wary of predation than smaller bat species. *Eonycteris spelaea* can even persist in limestone caves in Kuala Lumpur, Malaysia, one of the largest cities in southeast Asia [38, 43]. Thus, this bat species may actually benefit from some land use changes, as many bat-pollinated plant species are intentionally cultivated by humans (e.g., durian and sator) and others thrive in the sunny, open habitats maintained by humans (e.g., Indian trumpet flower and wild banana).

In contrast, we observed significant declines for two other important bat pollinators, *M. minimus* and *M. sobrinus* (Figs. 2, 3). Previous work has shown that *Macroglossus* bats are the main pollinators of banana and mangrove apple flowers in southern Thailand [45, 60]. The findings of this study show that the number of *M. sobrinus* netted at banana flowers dropped 80% when comparing data from 2011–2014 and 2019–2021 (Fig. 3). Similarly, the number of *M. minimus* bats netted at mangrove apple flowers dropped 81% between 2011–2014 and 2019–2021 (Fig. 3). Several factors have likely contributed to these declines. We hypothesize that *Macroglossus* bats are more affected than *E. spelaea* is by ongoing habitat loss and degradation, since *Macroglossus* species roost in vegetation [57, 73] while *E. spelaea* roosts in caves [1]. Kingston [33] studied insectivorous bats in Malaysia and also reported cave-roosting species to be more resilient to forest loss and degradation than foliage-roosting species. *Macroglossus minimus* bats may be particularly affected by land conversion as they primarily roost in mangroves [57], and Thailand has already lost over half of its mangrove forest cover [9]. Moreover, *Macroglossus* bats are less than half the size of *E. spelaea* [21] and have shorter lifespans [7] and much smaller foraging ranges; average home range has been estimated to be 5.8 ha for *M. minimus* [73] and 518 ha for *E. spelaea* [2]. Since *E. spelaea* has a long life span and foraging range, it can presumably visit the same resource-rich sites for several years, while smaller bats with shorter life spans and foraging ranges may be more affected by habitat change and more likely to relocate. Winfree et al. [72] also found that dietary specialists, such as *Macroglossus* bats, are more sensitive to changes in land use than are dietary generalists, such as *E. spelaea*. Finally, *Macroglossus* bats appear to be less tolerant of human disturbance, which may make them less likely to roost in and forage at cultivated banana plants, and shrinking areas of natural habitat may contribute to the significant declines observed in this study.

Pollinator declines are troubling not only for the pollinator species themselves, but also for the plant species that depend on them for pollination. The observed declines in the number of *Macroglossus* bats caught at banana and mangrove apple flowers are likely to affect plant reproductive success given that the former are primarily pollinated by *M. sobrinus* and the latter by *M. minimus* [60]. While cultivated bananas are parthenocarpic, wild bananas require pollination to reproduce [5], and wild bananas can be an important source of genetic diversity, particularly given the susceptibility of many clonal banana cultivars to disease [16]. Reduced pollinator visitation to mangrove apple flowers is of even greater concern given that these ecologically important species depend on pollinators to reproduce, particularly for the critically endangered *S. griffithii* [45] and the near threatened *S. ovata* [44]. Thus, the observed reduction in pollinator visitation, combined with intensive mangrove deforestation and other land conversion in southern Thailand [64], is likely to have substantial effects on the reproductive success of these plant species and further research is necessary to monitor changes in wild banana and mangrove apple reproduction.

It is important to note that the observed declines in the number of bats netted at floral resources does not necessarily indicate declines in population size. One possible alternative explanation is that differences in flowering intensity across years may be responsible for the observed declines. We think this explanation is unlikely given that the two plant species where declines were observed (banana and mangrove apple) exhibit steady-state flowering throughout all or most of the year, and flowering intensity appeared similar across study years (A. Stewart, pers. obs.); however, it is possible that other changes in food resources (e.g., nectar volume or concentration) are influencing bat foraging. Another potential explanation could be that foliage-roosting bats such as *M. minimus* and *M. sobrinus* are moving deeper into forests, farther away from human activity, and thus were netted less often at the human-cultivated plants (e.g., durian and sator) and sun-loving species (e.g., Indian trumpet flower and wild banana) that were the focus of this study. This possibility could also explain the significant declines in *C. sphinx* netted at banana flowers and *C. horsfieldii* netted at Indian trumpet flowers (Fig. 3), given that *Cynopterus* bats also roost in foliage [14]. However, contrary to this hypothesis, a recent study in Indonesia found that *M. minimus* bats were twice as abundant in plantations than in forests because of the relative abundance of banana plants [70], and *Cynopterus* bats are generally reported to be tolerant of human disturbance [14, 15, 39]. This explanation is still troubling given current deforestation rates in Thailand [17, 64], even in national parks and other protected areas [18], which means that even natural refuges are shrinking. While current IUCN Red List reports state that *Eonycteris*, *Macroglossus*, and *Cynopterus* bats are of Least Concern, the findings of this research indicate that population studies of nectarivorous bats in southeast Asia are urgently needed for updated species conservation assessments.

### Supplementary Information

**Supplementary Material 1.** 

## Data Availability

The data used in this study are openly available in Mendeley Data at https://data.mendeley.com/datasets/cmkhjnn29d/1 (10.17632/cmkhjnn29d.1).
