# Supplementary material for: Bat pollinators: a decade of monitoring reveals declining visitation rates for some species in Thailand
Source: Zoological Lett. 2024 Mar 2;10:5. doi: 10.1186/s40851-024-00228-x (PMC10908063; doi:10.1186/s40851-024-00228-x)
Supplement: Supplementary file 1 — Supplementary Material 1. [file 40851_2024_228_MOESM1_ESM.pdf]

Bat pollinators: a decade of monitoring reveals declining visitation rates for some species in Thailand

Supplementary Table 1. Mist-netting effort (total number of mist-net hours; mnh) and net height (mean height ± SE) at plant study species across study years. Note: Sampling was not conducted at *Durio zibethinus* in 2011 or 2019, or at *Sonneratia* species in 2011 or 2021, and net heights were only recorded for three study species (years 2013-2014 and 2019-2021).

		**2011**	**2013**	**2014**	**2019**	**2020**	**2021**
**Mist-netting effort (mnh)**	*Durio zibethinus*	n/a	78	72	n/a	62	50
	Musa acuminata	48	60	66	52	30	53
	Oroxylum indicum	20	54	24	24	24	25
	Parkia speciosa	51	48	42	44	39	35
	Sonneratia spp.	n/a	84	60	37	40	n/a
**Net height (m)**	*Musa acuminata*	n/a	3.29±0.29	2.78±0.15	2.33±0.21	3.00±0.41	2.88±0.52
	Oroxylum indicum	n/a	8.80±0.49	7.00±0.71	8.80±0.58	8.50±1.18	8.40±1.37
	Parkia speciosa	n/a	7.60±1.17	8.00±1.05	7.80±1.39	6.00±0.00	7.67±0.67

Supplementary Table 2. Linear mixed model results (F-ratio, degrees of freedom (df), and p-values (P)) showing the effect of two main factors (bat species and time period) and their interaction on the number of bats netted per hour at the flowers of five bat-pollinated plant taxa. Significant predictors are highlighted in bold.

	Predictor	F-ratio	df	P
Durio zibethinus	Bat species × Time period	0.735	6	0.622
	**Bat species**	39.58	6	<0.001
	Time period	0.073	1	0.790
Musa acuminata	**Bat species × Time period**	3.631	6	0.002
	**Bat species**	21.21	6	<0.001
	Time period	1.594	1	0.214
Oroxylum indicum	**Bat species × Time period**	2.311	6	0.036
	**Bat species**	31.19	6	<0.001
	Time period	0.105	1	0.748
Parkia speciosa	Bat species × Time period	1.483	6	0.185
	**Bat species**	79.00	6	<0.001
	Time period	0.185	1	0.669
Sonneratia spp.	**Bat species × Time period**	5.304	6	<0.001
	**Bat species**	26.05	6	<0.001
	Time period	0.587	1	0.453

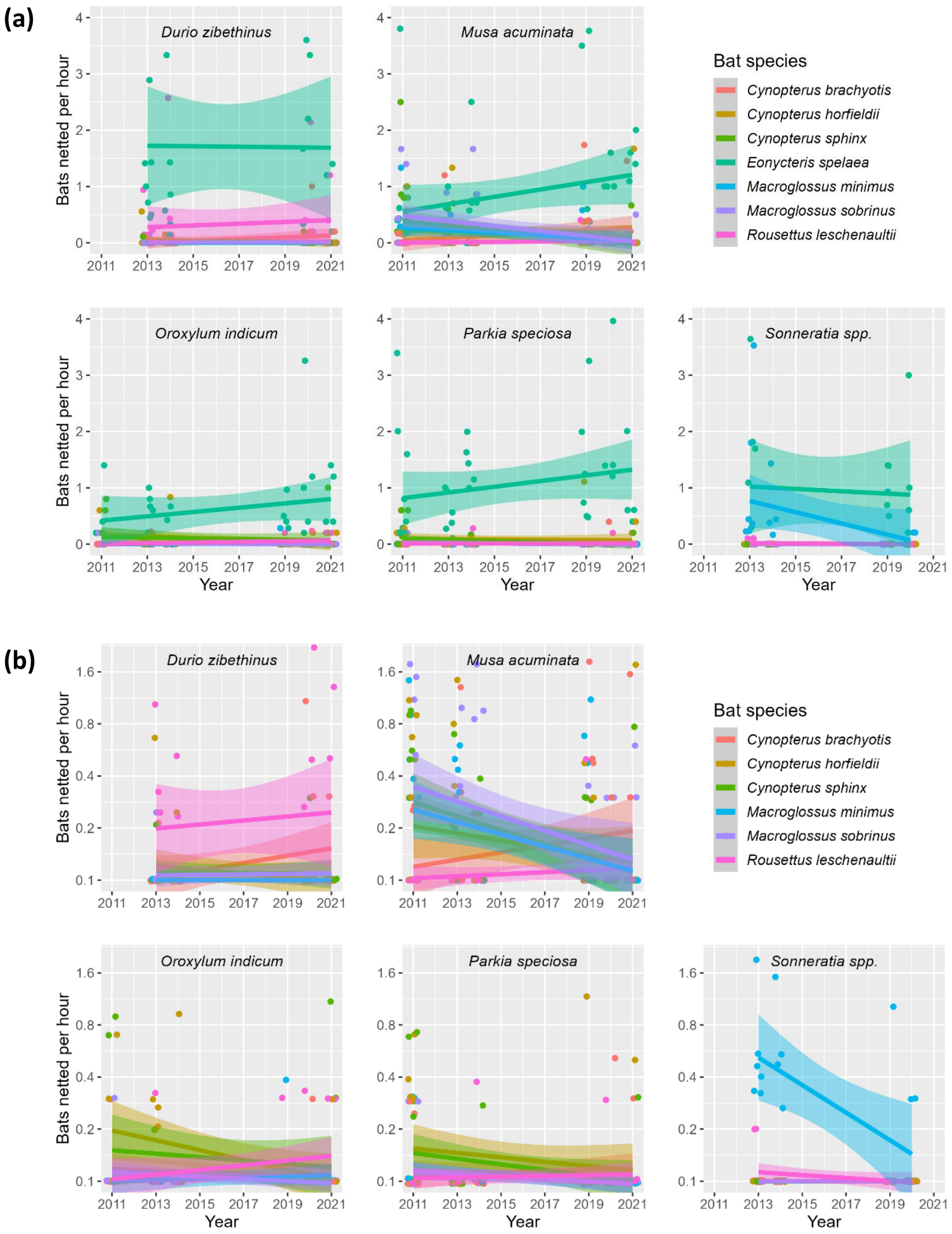

Supplementary Figure 1. Results of LMM showing the number of bats netted per hour at five bat-pollinated plant taxa between 2011-2021 for (a) all seven flower-visiting bat species and (b) six of the bat species (*Eonycteris spelaea* excluded to improve visualization of the other species). Points show actual data; lines and shaded areas show linear regression lines and 95% confidence intervals, respectively. Note: The y-axis for (b) is log-scaled.

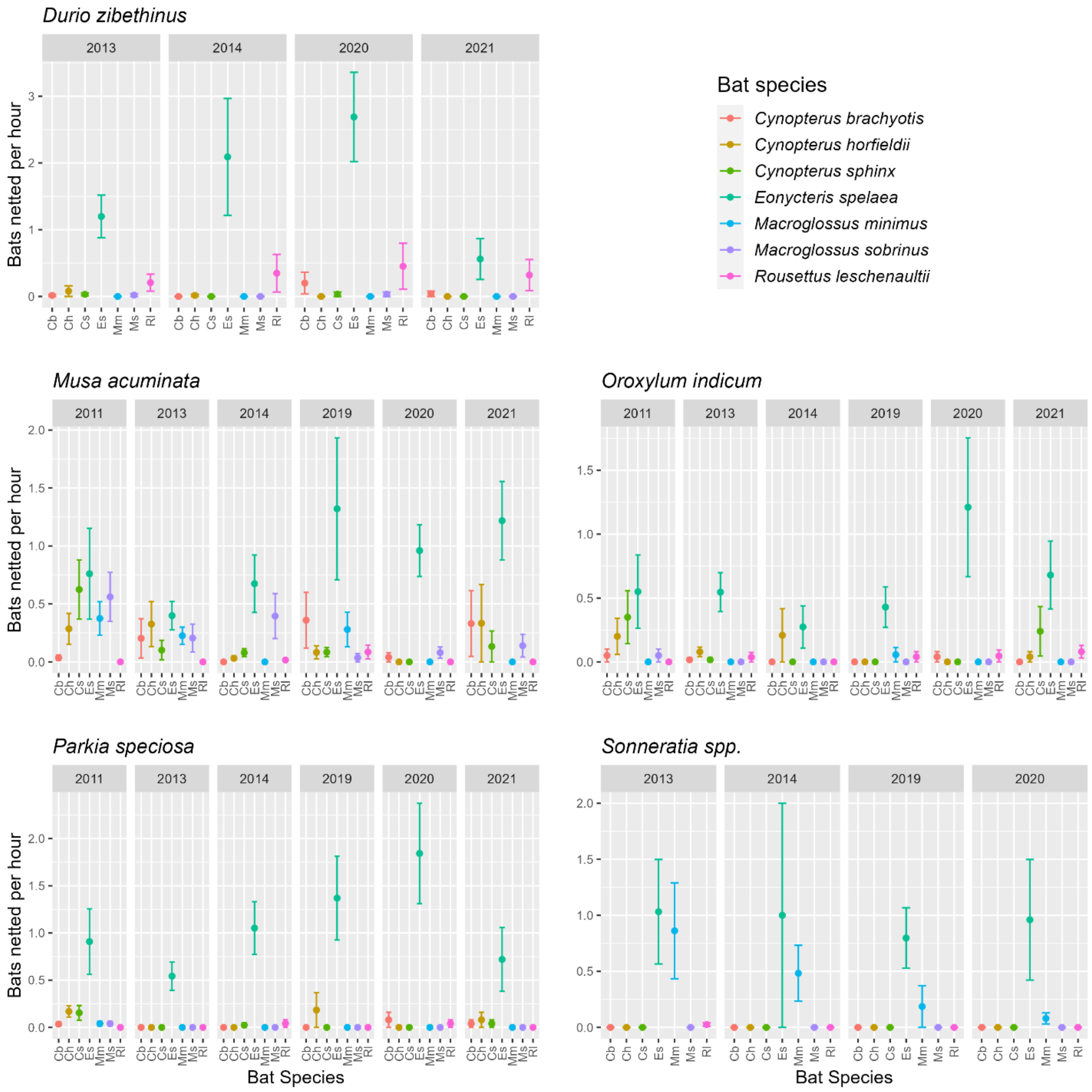

Supplementary Figure 2. The number of bats netted per hour (mean ± SE) for seven nectar-feeding bat species at five bat-pollinated plant taxa between 2011-2021.

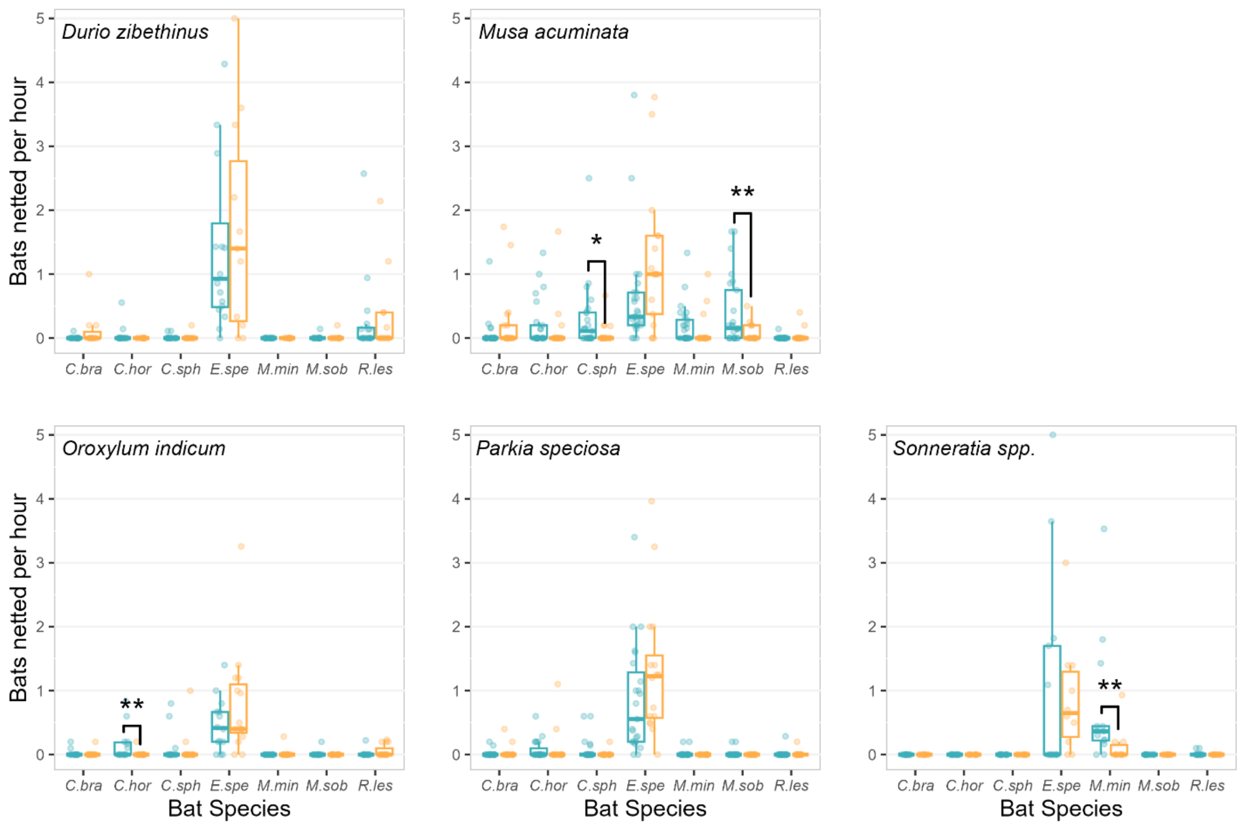

Supplementary Figure 3. A comparison of the number of bats netted per hour for seven nectar-feeding bat species at five bat-pollinated plant taxa between 2011-2014 (teal) and 2019-2021 (orange). Boxplots summarize the data for each time period; each jittered point represents a single night. Significant differences between the two time periods are denoted with asterisks (one asterisk, $P < 0.05$; two asterisks, $P < 0.01$). Abbreviations: *C. bra*, *Cynopterus brachyotis*; *C. hor*, *Cynopterus horsfieldii*; *C. sph*, *Cynopterus sphinx*; *E. spe*, *Eonycteris spelaea*; *M. min*, *Macroglossus minimus*; *M. sob*, *Macroglossus sobrinus*; *R. les*, *Rousettus leschenaultii*.
